# Editorial: When Chemistry Meets Biology – Generating Innovative Concepts, Methods and Tools for Scientific Discovery in the Plant Sciences

**DOI:** 10.3389/fpls.2016.00076

**Published:** 2016-02-08

**Authors:** Erich Kombrink, Markus Kaiser

**Affiliations:** ^1^Chemical Biology Laboratory, Max Planck Institute for Plant Breeding ResearchKöln, Germany; ^2^Department of Chemical Biology, Faculty of Biology, Center for Medical Biotechnology, University of Duisburg-EssenEssen, Germany

**Keywords:** agricultural biotechnology, bioactive small molecule, chemical biology, chemical genetics, high-throughput screening, phytohormones, plant defense, plant–pathogen interactions

The term “chemical biology” is commonly associated with research at the interface of chemistry and biology, as revealed by a recent census amongst scientists working in the field (Anonymous, 2015). Distinctively, as a truly interdisciplinary science, chemical biology combines scientific concepts and experimental approaches from both of its parent fields to understand molecular mechanisms in complex biological systems. New chemical tools and technologies are developed to dissect, visualize and manipulate biological processes or pathways and, conversely, studying biological systems may foster the development of new chemical principles. Although chemical biology has so far found most applications in pharmaceutical drug discovery, the search for novel bioactive small molecules that produce phenotypes in plants is also an established strategy in the agrichemical industry. In these approaches, the primary focus was laid on the discovery and improvement of herbicides, pesticides and other agriculturally useful compounds.

Upon recognition of the opportunities that bioactive small molecules may provide to basic plant biology research, in particular in combination with genetic strategies, the research field of plant “chemical genetics” emerged and has grown substantially over the past decade. Essentially, small molecules are utilized to generate recognizable phenotypes in a manner that is analogous to forward mutation genetics. However, chemical genetics has the potential to circumvent inherent problems of classical, forward genetics, such as lethality, pleiotropy or redundancy of gene functions because small molecules can be applied in a conditional, dose-dependent and reversible manner. The persistent challenge remains to identify the cognate target of such bioactive chemicals to discover the genes, proteins or pathways that are responsible for a given phenotype. Likewise, chemicals can be combined with other profiling technologies, such as genomics, proteomics or metabolomics. There are now clear examples of important, fundamental discoveries originating from plant chemical biology/genetics that demonstrate the power of this experimental approach.

The current issue on plant chemical biology provides a snapshot of the field, comprising review articles, perspectives and original research articles that both novices and experts may find useful. In their perspective article, Hicks and Raikhel focus on successful applications and the current challenges that the field of plant chemical biology/genetics faces as it matures. The discovery of novel bioactive chemicals is generally achieved by screening of compound collections (so-called chemical libraries), using robust and reliable phenotypes. The design and execution of such screening campaigns is outlined in a review article by Serrano et al. Here, in particular the newcomer will find useful recommendations that will help to avoid common pitfalls. In addition, recent success stories of plant chemical biology are highlighted, which may serve as teaching examples for implementation of future chemical biology projects. Along the same lines, in an original research article, Halder and Kombrink describe a facile bioassay for quantifying β-glucuronidase (GUS) activity *in situ*, which may turn out as a useful screening tool. Importantly, the methodology can be adopted for any transgenic *Arabidopsis* line harboring an inducible (or repressible) GUS reporter, and such lines are available for numerous developmental stages or signaling pathways. Once a chemical screening campaign has provided a new bioactive compound, the ultimate goal is to identify its mode of action and molecular target (or targets). Dejonghe and Russinova discuss in their review article different strategies for direct target identification, the current ones as well as the emerging ones, which have not yet found broad application in plant biology. Despite all recent progress, this still remains the most challenging, laborious and time-consuming step of chemical biology projects.

A recurring theme of plant chemical biology has been the search for, and application of bioactive small molecules in plant hormone signaling. In this sector a large assortment of chemicals has been identified and used as agonists or antagonists of phytohormones and thereby provided new insights into plant hormone biology. Two review articles by Rigal et al. and Fonseca et al. summarize some prominent examples of using chemical biology/genetic strategies in plant hormone research. Redundancy is largely avoided, as the former article focuses on signaling mediated by abscisic acid (ABA), salicylic acid (SA), auxin (IAA), cytokinin (CK), and brassinosteroids (BR), while the latter covers jasmonate (JA) signaling as well as phytohormone homeostasis, transport and hormonal crosstalk. In addition, Nakamura and Asami provide a focused review on chemical regulation of strigolactone (SL) signaling. Strigolactones have recently attracted much attention, as they are multifunctional molecules that not only act as phytohormones, inhibiting shoot branching, but also serve as rhizospheric communication signals between plant and symbiotic fungi and/or parasitic plants from the *Striga* and *Orobanche* genera. Identification or design of inhibitors of SL biosynthesis or SL receptors is a potential method to control these devastating and agronomically important root parasites. Finally, Almeida-Trapp et al. describe in an original research article the development and validation of an analytic method for quantification of six phytohormones that are frequently associated with stress responses, IAA, ABA, SA, JA, jasmonoyl-isoleucine (JA-Ile), and 12-oxo-phytodienoic acid (OPDA). Such a critically evaluated and validated method is obviously important for all plant hormone related work allowing direct comparison of hormone levels established in different laboratories.

A second favorite topic of plant chemical biology research has been plant–pathogen interactions and plant immune responses. Naturally occurring small molecules such as toxins produced by pathogens or phytoalexins produced by plants upon infection serve to intercept with growth and development of plants and pathogens, respectively. Other, not-so-small molecules (i.e., peptides) are involved in the initial perception of pathogen invasion by specific plant receptor-like kinases. Mott et al. discuss in their review the diverse roles of apoplastic molecules (peptides and small molecules) in modulating plant–pathogen interactions. A different view on such interactions is provided by Bektas and Eulgem who discuss in their review the function of synthetic elicitors that induce plant defense responses, but are distinct from known natural elicitors of plant immunity. A large variety of such compounds has been identified through screening efforts or targeted synthesis as analogs of natural compounds such as salicylic acid. They are attractive for basic research, allowing functional dissection of the plant immune system, as well as for applied purposes, as they can protect crop plants from diseases. Stokes and McCourt develop the applied aspect of chemicals in biological systems (here chemistry and agricultural biotechnology) a step further by predicting a future trend toward “personalized” agriculture, which essentially means to develop highly selective and species-specific herbicides and growth regulators. Indeed, recent success stories in plant chemical biology demonstrate that the corresponding technologies and tools are available. Thus, the development of tailored chemicals that modify traits of crop species or target specific classes of weeds or pests by collaboration of applied and academic research groups (in analogy to the current drug discovery process) may provide a bright future for plant chemical biology.

## Author contributions

EK served as editor of the research topic “When Chemistry Meets Biology – Generating Innovative Concepts, Methods and Tools for Scientific Discovery in the Plant Sciences” and wrote this editorial. MK served as editor of the research topic “When Chemistry Meets Biology – Generating Innovative Concepts, Methods and Tools for Scientific Discovery in the Plant Sciences” and wrote this editorial.

### Conflict of interest statement

The authors declare that the research was conducted in the absence of any commercial or financial relationships that could be construed as a potential conflict of interest.
